# The correlation between embryo rescue and hormonal changes in seedless grapes

**DOI:** 10.3389/fpls.2024.1460886

**Published:** 2024-09-18

**Authors:** Guirong Li, Kaiwei Li, Yihai Lu, Xiucai Fan, Ling Wang

**Affiliations:** ^1^ School of Horticulture and Landscape Architecture, Henan Institute of Science and Technology, Xinxiang, Henan, China; ^2^ Henan Province Engineering Research Centers of Horticultural Plant Resource Utilization and Germplasm Enhancement, Xinxiang, Henan, China; ^3^ Zhengzhou Fruit Research Institute, Chinese Academy of Agricultural Sciences, Zhengzhou, Henan, China

**Keywords:** embryo rescue breeding technology, embryonic development, hormone, seedless grape, *in vitro*

## Abstract

After normal pollination and fertilization of pseudoparthenocarpic seedless grapes, their embryos often stop developing due to certain developmental factors, resulting in embryo abortion. Hybrid breeding using seedless grapes as the maternal parent requires embryo rescue breeding technology. This technology plays a crucial role in seedless grape breeding. Although previous studies have extensively explored this technology, knowledge regarding its impact on embryo abortion and the effectiveness of rescue techniques remains limited. This study aimed to investigate the correlation between embryo rescue and hormonal changes in seedless grapes. Four Eurasian seedless grape cultivars, “Thompson Seedless,” “Flame Seedless,” “Heshi Seedless,” and “Ruby Seedless,” were selected for examination. We investigated endogenous hormone levels, including indole-3-acetic acid (IAA), gibberellic acid (GA_3_), and abscisic acid (ABA), in both berries and *in vitro* ovules during the most suitable embryo rescue time for these cultivars. Based on the observed fluctuations in endogenous hormone levels and previous research findings, appropriate doses of exogenous hormones, such as IAA, GA_3_, and ABA, were applied during seedless grape embryo rescue. The results indicated significant differences in endogenous hormone levels between berries with varying ovule counts of the same cultivar and ovules cultured *in vitro*, suggesting a hormonal influence on ovule abortion and embryo development in seedless grapes. Further research has identified effective ratios of exogenous hormones: 30 mg·L^–1^ IAA + 30 mg·L^–1^ ABA for berry ovule development, 1.0 mg·L^–1^ IAA + 2.0 mg·L^–1^ 6-benzylaminopurine (6-BA) + 1.0 mg·L^–1^ GA_3_ + 1.0 mg·L^-1^ ABA for *in vitro* ovule development, and 1.0 mg·L^–1^ IAA + 2.0 mg·L^–1^ 6-BA + 1.0 mg·L^–1^ GA_3_ for embryo germination and seedling formation. In summary, hormonal changes significantly influence ovule and embryo development and are closely related to seedless grape embryo rescue breeding. This study deepened our understanding of the correlation between seedless grape embryo rescue and hormonal changes. It also resulted in the successful production of a batch of embryo rescue seedlings, further improving embryo rescue breeding technology and providing new germplasm materials for developing new seedless grape cultivars.

## 1 Introduction

Grapes (*Vitis vinifera* L.) are an essential fruit tree crop cultivated globally, valued for their nutrient-rich fruits and versatile applications such as wine production. Seedless cultivars are particularly highly adapted among grape cultivars due to their seedless characteristics. However, challenges during embryonic development hinder further increases in quality and yield. Abnormal embryo development can result in reduced fruit size, quality, and resistance, thereby reducing the economic value of seedless grapes (Li et al., 2019). In recent years, embryo rescue for seedless grapes has emerged as an approach to improve the efficiency of seedless grape breeding and obtain new germplasm. Understanding the mechanism of embryo abortion, employing suitable rescue techniques, and obtaining rescue germplasm are crucial for advancing novel seedless grape breeding (Zhu et al., 2022).

Studying the effects of hormonal changes on ovule and embryo development during the embryonic growth of seedless grapes is crucial. By applying appropriate exogenous hormones to fruits and *in vitro*-cultured ovules, the seedling rate of embryo-rescued seedlings can be increased. This approach can significantly advance seedless grape embryo rescue breeding technology. Previous studies have demonstrated a crucial regulatory role of plant hormones in the development of grape berries, such as indole-3-acetic acid (IAA) (Santa-Catarina et al., 2006; Hossain et al., 2021; Karami et al., 2023), 6-benzylaminopurine (6-BA) (Liu et al., 2015; Khoshandam et al., 2017), gibberellic acid (GA_3_) (Yan et al., 2020; Şenay et al., 2022), and abscisic acid (ABA) (Santa-Catarina et al., 2006; Vahdati et al., 2008). However, the specific impact of these hormones on seedless grape embryo rescue remained underexplored. Li et al. (2021) used various hormones such as IAA, ABA, and GA_3_ to explore co-expressed genes related to hormone regulation during seed development. They further conducted an in-depth investigation into the mechanisms of embryo abortion.

Previous studies also investigated the changes in hormone levels and ratios during the embryonic development of seedless grapes. The endogenous IAA and GA_3_ levels were higher in seedless grapes than in seed-bearing grapes, suggesting that the changes in endogenous hormone levels might contribute to embryo abortion (Baydar and Harmankaya, 2005). Li et al. (2020c) determined the most suitable embryo rescue time by analyzing the decrease in the ratio of endogenous hormones, [GA_3_ + IAA]/ABA, on the 36th day. Zhu et al. (2022) conducted research using hormones and found that the optimal culture medium for transforming deformed seedlings into normal seedlings was 2 × MS medium containing 0.2 mg·L^−1^ 6-BA, 0.1 mg·L^−1^ IAA, and 1.6 mg·L^−1^ ZnSO_4_. Zhu et al. (2023) compared the seedless cultivar “Jingxiu” with “Qinxiu” and found that the IAA and GA_3_ levels in the ovules of “Qinxiu” were lower, whereas the ABA level was higher. Additionally, the ratios of (IAA + GA_3_)/ABA and (IAA + ZT + GA_3_)/ABA were lower in “Qinxiu,” which might be one of the main reasons for embryo abortion. Seeds require different types and concentrations of exogenous hormones in different developmental stages. Improper use of exogenous hormones in terms of type or concentration could affect the hormone level in fruits and seeds, leading to hormonal imbalance and adversely affecting embryo development, ultimately leading to abortion and a decrease in the fresh weight of ovules (Amaral et al., 2001; Zhu et al., 2022; Zhu et al., 2023).

Previous studies on the correlation between embryo rescue and hormonal changes in seedless grapes are limited. Most studies focused on the correlation between the endogenous hormone level and embryo development, with fewer studies exploring the role of exogenous hormones. Differences in hormonal changes were observed among different genotypes of seedless grapes. Studying the correlation between embryo rescue and hormones could offer a theoretical basis for promoting embryo development in seedless grape breeding. Additionally, although efficient ovule development required advanced embryonic development, identifying the optimal sampling time for each cultivar was crucial for successful embryo rescue (Jiao et al., 2018). This step was essential for cultivating novel seedless grapes using embryo rescue breeding technology (Li et al., 2015; Xu et al., 2022). Therefore, this study investigated the correlation between embryo rescue and hormonal changes in seedless grapes. It revealed the changes in endogenous hormone levels in the fruits and ovules of different seedless grape cultivars during the most suitable embryo rescue time. Combining these hormonal changes with previous findings, the study also examined the effects of exogenous hormones on embryo rescue breeding. Determining the optimal combination of exogenous hormones is crucial for berry ovule development, *in vitro* ovule growth, and embryo germination. The study involved inoculating ovules with different exogenous hormone ratios, measuring their development and germination rates and obtaining a batch of seedless grape seedlings through embryo rescue. This study further explored the correlation between hormonal changes and embryo rescue breeding in seedless grapes, improved embryo rescue breeding techniques, and provided a theoretical basis for improving seedling rates. It also offered scientific support for the breeding and production of seedless grapes.

## 2 Materials and methods

### 2.1 Test materials and their treatment

Test materials: four naturally pollinated seedless grape cultivars, “Thompson Seedless,” “Flame Seedless,” “Heshi Seedless,” and “Ruby Seedless,” were selected and sampled at the most suitable embryo rescue time. Specifically, the samples were taken after 37 days for “Thompson Seedless,” 45 days for “Flame Seedless,” 60 days for “Heshi Seedless,” and 65 days for “Ruby Seedless” (Li et al., 2014, 2024). The grape materials were obtained from the grape germplasm resource nursery at the Northwest University of Agriculture and Forestry Science and Technology.

Material treatment: eight clusters from each of the four naturally pollinated cultivars (Thompson Seedless, Flame Seedless, Heshi Seedless, and Ruby Seedless) were randomly selected, placed in a plastic foam box with ice, and quickly refrigerated. These clusters were then transported back to the laboratory. The fruits were counted and classified into four categories: no ovule, one ovule, two ovules, and three ovules. For each category, 90 fruits of each type were randomly selected and divided into three groups of 30 fruits each. After 8 weeks of *in vitro* culture, the ovules from each category were examined, peeled off, and further classified into two types: those with embryos and those without. Sixty ovules were selected from each type, with 20 ovules in each group, totaling three groups. Initially, the ovules were soaked in distilled water to remove any impact of the attached medium on the results, followed by rinsing with deionized water three times. The removed ovules and ovules without embryos were processed and mixed separately, whereas the extracted embryos were still inoculated and cultured. Finally, the samples were frozen in liquid nitrogen and stored in a refrigerator at –80°C (Li et al., 2024).

### 2.2 Test methods

#### 2.2.1 Determination of endogenous hormone levels in seedless grape berries

After preserving the treated materials, 1 g of samples was obtained from each type (no ovule, one ovule, two ovules, and three ovules) of grape berries by grinding. The samples were ground with 80% pre-cooled methanol containing 1 mmol·L^–1^ di-*tert*-butyl-*p*-cresol in an ice bath under low-light conditions. Then, IAA, GA_3_, and ABA levels were determined using the enzymic-linked immunosorbent assay method described by Wu et al. (1988). Endogenous hormone levels in the berries of the four seedless grape cultivars were determined three times for each category.

#### 2.2.2 Determination of endogenous hormone levels in *in vitro* ovules of seedless grapes

The endogenous hormones from two categories (with and without embryo) of *in vitro* ovules from seedless grapes were extracted, and their levels were determined using the same method as for the berries. Endogenous hormone levels in the *in vitro* ovules of four seedless grapes were also determined.

#### 2.2.3 Spraying exogenous hormones on seedless grape berries

Exogenous hormones (IAA, GA_3_, and ABA) were bought from the Shanghai Chemical Reagent Company. Initially, a small amount of ethanol was added to aid dissolution, followed by dilution with water. The formulation of the prepared exogenous hormone solution was determined, as depicted in **Table 1**, using an orthogonal experimental design (three factors and two levels, resulting in four treatments), generally prepared on the same day as the ear treatment. We carried out a preliminary experiment referring to the results of Finkelstein et al. (1985) and Şenay et al. (2022). We found that the effect of spraying exogenous hormones was the best at 30 mg·L^–1^. Therefore, the two levels we designed were 0 and 30 mg·L^–1^.

**Table 1:** L4(2^3^) orthogonal design table of exogenous hormone (mg·L^-1^).

Treatment^a^	IAA	GA_3_	ABA
EH1	0	0	0
EH2	0	30	30
EH3	30	0	30
EH4	30	30	0

a Treatments of different concentrations of exogenous hormones on seedless grapes were divided into four groups: EH1, EH2, EH3, and EH4 (endogenous hormone abbreviated as EH).

The application method involved spraying the ears using a handheld spray gun between 18:00 and 21:00 when the temperature ranged from 22°C to 30°C, ensuring thorough wetting of the entire ear. One tree was designated as the experimental group, and five ears in the same developmental stage were selected for chemical spraying as a treatment. The treatment involved spraying once every other week, repeated three times. Ears that were not sprayed served as the control group.

#### 2.2.4 Culturing seedless grape ovules *in vitro* using exogenous hormones

**Figure 1:**
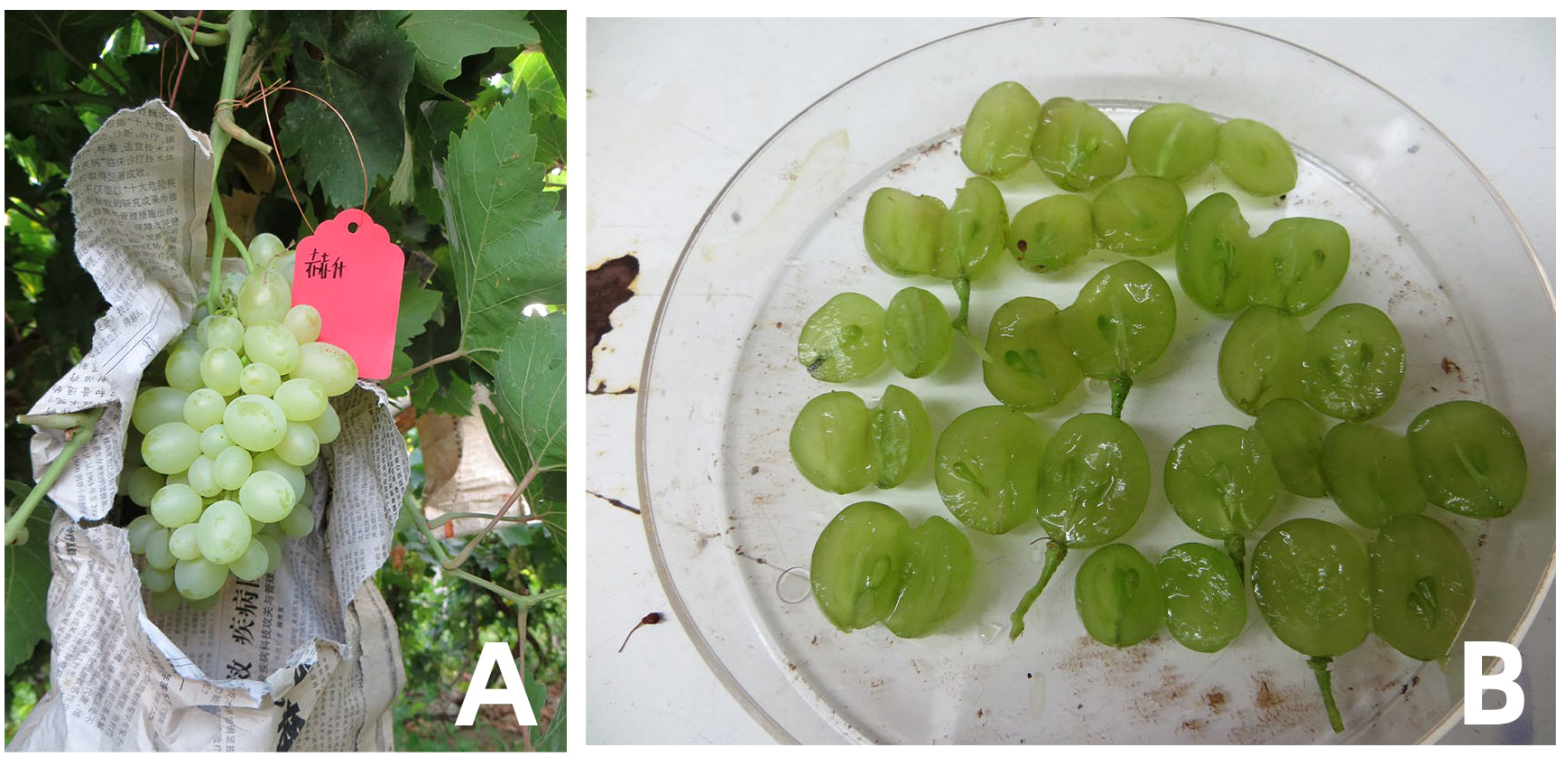
Sampling of the *in vitro* ovules of “Heshi Seedless” grapes. **(A)** A “Heshi Seedless” grapefruit after natural pollination for 60 days. **(B)** Ovules of “Heshi Seedless” grapes naturally pollinated.

In this stage, “Heshi Seedless”, as the female parent used in later stages of seedless grape hybrid breeding, was selected as the experimental material for the study. A certain number of naturally pollinated ovules (ovule length ≥2 cm) (**Figure 1**) were selected and inoculated into Erlenmeyer flasks (100 ml) containing a solid-liquid double-layer medium. The solid medium used was a modified Minimal Medium 3 (MM3), which included the macro- and micro-elements of MM3, ferric salt from MS, and organic components from Eriksson medium (ER). This medium was further supplemented with 0.05% (w/v) casein hydrolysate (CH), 6% (w/v) sucrose, 0.3% (w/v) activated charcoal, and 0.7% (w/v) agar, with the pH adjusted to 6.0. For the liquid medium, the ER medium was employed (Li et al., 2014). Various hormone ratios were added to the solid-liquid medium following an orthogonal study design (four factors and three levels, totaling nine treatments). The specific formulations are presented in **Table 2**. Pre-experiments were conducted based on the findings of previous studies (Li, et al., 2020a; Tang et al., 2009b), revealing significant effects of exogenous hormones at concentrations of 1.0 and 2.0 mg·L^–1^. Consequently, three levels were designed: 0, 1.0, and 2.0 mg·L^–1^. The development rate of embryos was observed and recorded.

**Table 2:** L9(3^4^) orthogonal design table of exogenous hormones (mg·L^-1^).

Treatment^a^	IAA	6-BA	GA_3_	ABA
H1	0.0	0.0	0.0	0.0
H2	0.0	1.0	2.0	1.0
H3	0.0	2.0	1.0	2.0
H4	1.0	1.0	1.0	0.0
H5	1.0	0.0	2.0	2.0
H6	1.0	2.0	0.0	1.0
H7	2.0	0.0	1.0	1.0
H8	2.0	1.0	0.0	2.0
H9	2.0	2.0	2.0	0.0

a Treatments of different concentrations of exogenous hormones on in vitro culture of seedless grapes were divided into nine groups: H1, H2, H3, H4, H5, H6, H7, H8, and H9 (hormone abbreviated as H).

#### 2.2.5 Germination and seedling formation of seedless grapes cultured *in vitro* with exogenous hormones

After 8 weeks of ovule culture *in vitro*, the developed embryos were inoculated on a Woody Plant Medium (WPM) solid medium (Zhu et al., 2021). Different hormone ratios were added, as outlined in **Table 2**. The rates of embryo germination and seedling formation were then observed and recorded.

### 2.3 Statistical analysis

For an analysis of endogenous hormone levels, the berries and *in vitro* ovules of each cultivar were divided into three groups (*n* = 3). The corresponding averages and standard deviations were calculated based on the results of three repetitions (*n* = 3). The data analysis was performed using one-way analysis of variance with GraphPad Prism 8 (GraphPad Software Inc., CA, USA). Significant differences were indicated by asterisks (^*^ *P* < 0.05 and ^**^ *P* < 0.01).

## 3 Results

### 3.1 Changes in endogenous hormone levels in berries and *in vitro* ovules of seedless grapes

#### 3.1.1 Changes in endogenous hormone levels in seedless grape berries

**Figure 2:**
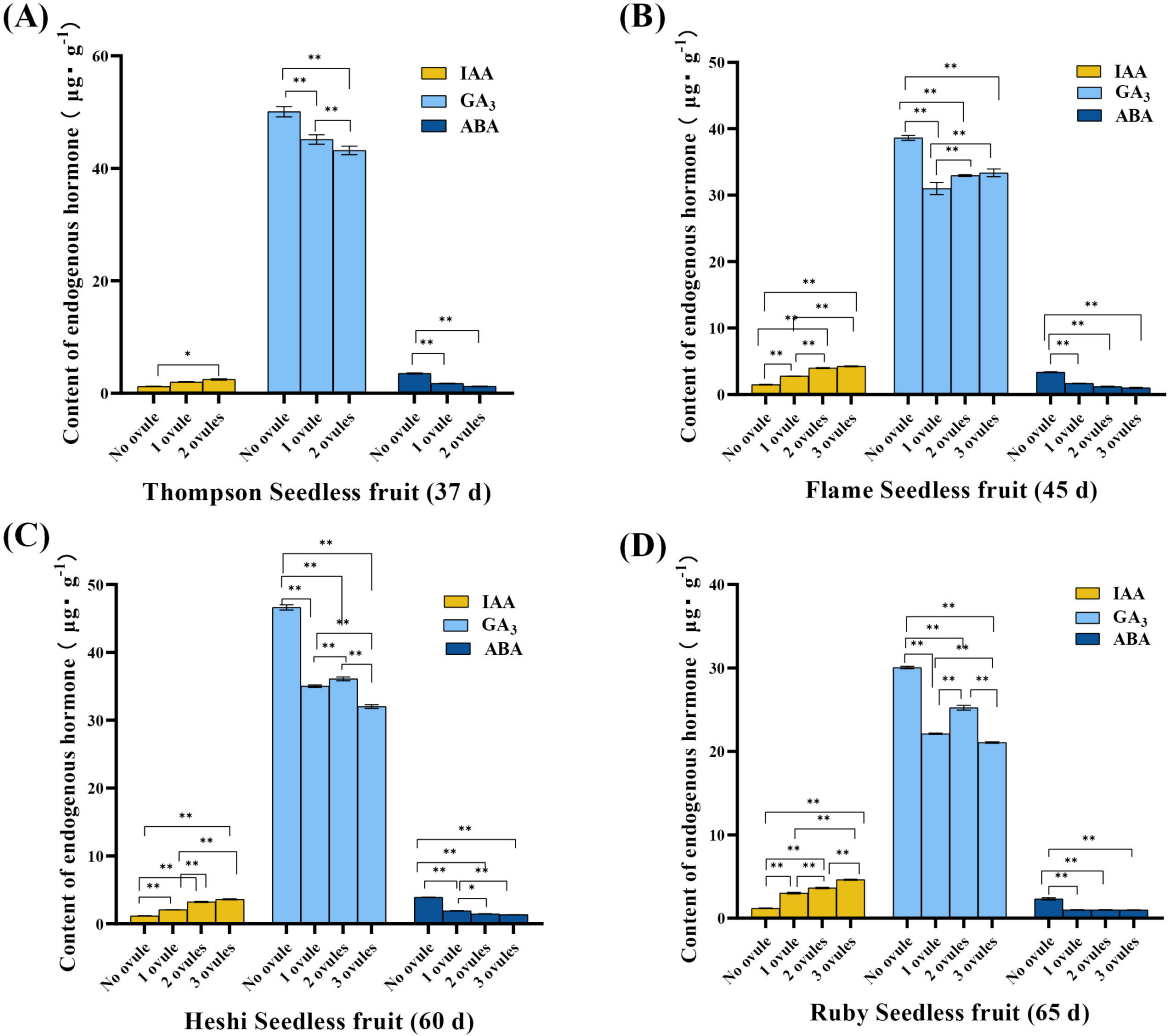
Endogenous hormones (µg·g^-1^) in berries of different samples of four seedless grapes (best embryo rescue time sampling). **(A)** “Thompson Seedless” grapes (37 days). **(B)** “Flame Seedless” grapes (45 days). **(C)** “Heshi Seedless” grapes (60 days). **(D)** “Ruby Seedless” grapes (65 days). The data are presented as mean ± SD, *n* = 3. ^*^ *P* < 0.05 and ^**^ *P* < 0.01 indicate significantly different values.

IAA level: In “Thompson Seedless” berries, the analysis revealed that the IAA level was significantly higher in berries with two ovules than in those without ovules. In “Flame Seedless” berries, the IAA level was significantly higher in berries with three ovules than those without ovules and one ovule. Moreover, the level was significantly higher in berries with two ovules than in those with fewer than two ovules, and the level was significantly higher in berries with one ovule than in those without ovules. In “Heshi Seedless” berries, the analysis showed that the IAA level was significantly higher in berries with three ovules than in those without ovules or one ovule, and the IAA level was significantly higher in those with two ovules than in those with fewer than two ovules. The IAA level was also significantly higher in berries with one ovule than in those without ovules. In “Ruby Seedless” berries, the IAA level was significantly higher in berries with three ovules than in those with fewer than three ovules, and the IAA level was significantly higher in berries with two ovules than in those with fewer than two ovules. The level was significantly higher in berries with one ovule than in those without ovules. The analysis data suggested that the higher IAA level was correlated with a significant number of ovules in these four seedless grapes, indicating that the elevated IAA level promoted ovule growth and development in berries. Conversely, the lower IAA level corresponded to a lack of ovules. This indicated that the reduced IAA level inhibited ovule growth to some extent, eventually leading to a decrease in their numbers until they disappeared (**Figure 2**).

GA_3_ level: In “Thompson Seedless” berries, the analysis revealed that the GA_3_ level was significantly higher in berries with two ovules than in those with fewer than two ovules. The level in one ovule was significantly higher than that without ovules. In “Flame Seedless” berries, the analysis showed that the GA_3_ level was significantly higher in berries with three ovules than in those with fewer than three ovules, and the level was significantly higher in berries with two ovules than in those with fewer than two ovules. The level was significantly higher in berries with one ovule than in those without ovules. In “Heshi Seedless” berries, the analysis indicated that the GA_3_ level was significantly higher in berries with three ovules than in those with fewer than three ovules, and the level was significantly higher in berries with two ovules than in those with fewer than two ovules. The level was significantly higher in berries with one ovule than in those without ovules. In “Ruby Seedless” berries, the analysis showed that the GA_3_ level was significantly higher in berries with three ovules than in those with fewer than three ovules, and the level was significantly higher in berries with two ovules than in those with fewer than two ovules. The GA_3_ level in one ovule was significantly higher than that without ovules. The analysis data showed that these four types of seedless grapes exhibited a low GA_3_ level when the number of ovules in the fruit was high, suggesting that a high GA_3_ level was not conducive to the growth and development of ovules in the berries. Conversely, when no ovule was present, the GA_3_ level was at its highest. This indicated that the high GA_3_ level in berries inhibited ovule growth to some extent, resulting in a reduction in their numbers until they disappeared (**Figure 2**).

ABA level: In “Thompson Seedless” berries, the analysis revealed that the ABA level was significantly higher in berries with two ovules and one ovule than in those without ovules. In “Flame Seedless” berries, the analysis showed that the ABA level was significantly higher in berries with three ovules, two ovules, and one ovule than in those without ovules. In “Heshi Seedless” berries, the analysis showed that the ABA level was significantly higher in berries with three ovules than in those with fewer than three ovules, and the level was significantly higher in berries with two ovules than in those with fewer than two ovules. The ABA level was significantly higher in berries with one ovule than in those without ovules. In “Ruby Seedless” berries, the ABA level was significantly higher in berries with three ovules, two ovules, and one ovule than in those without ovules. The analysis data showed that these four types of seedless grapes exhibited the low ABA level when the number of ovules in the fruit was high, indicating that the high ABA level was not conducive to the growth and development of ovules in the berries. Conversely, when no ovule was present, the ABA level was higher. This suggested that the high ABA level in berries inhibited ovule growth to some extent, resulting in a reduction in their numbers until they disappeared (**Figure 2**).

It was evident that these three endogenous hormones were closely associated with the development of drupe ovules, suggesting that a decrease in the IAA level and an increase in the GA_3_ and ABA levels might lead to a decrease in the number of ovules, potentially resulting in their eventual disappearance.

#### 3.1.2 Changes in endogenous hormone levels in *in vitro* ovules of seedless grapes

**Figure 3:**
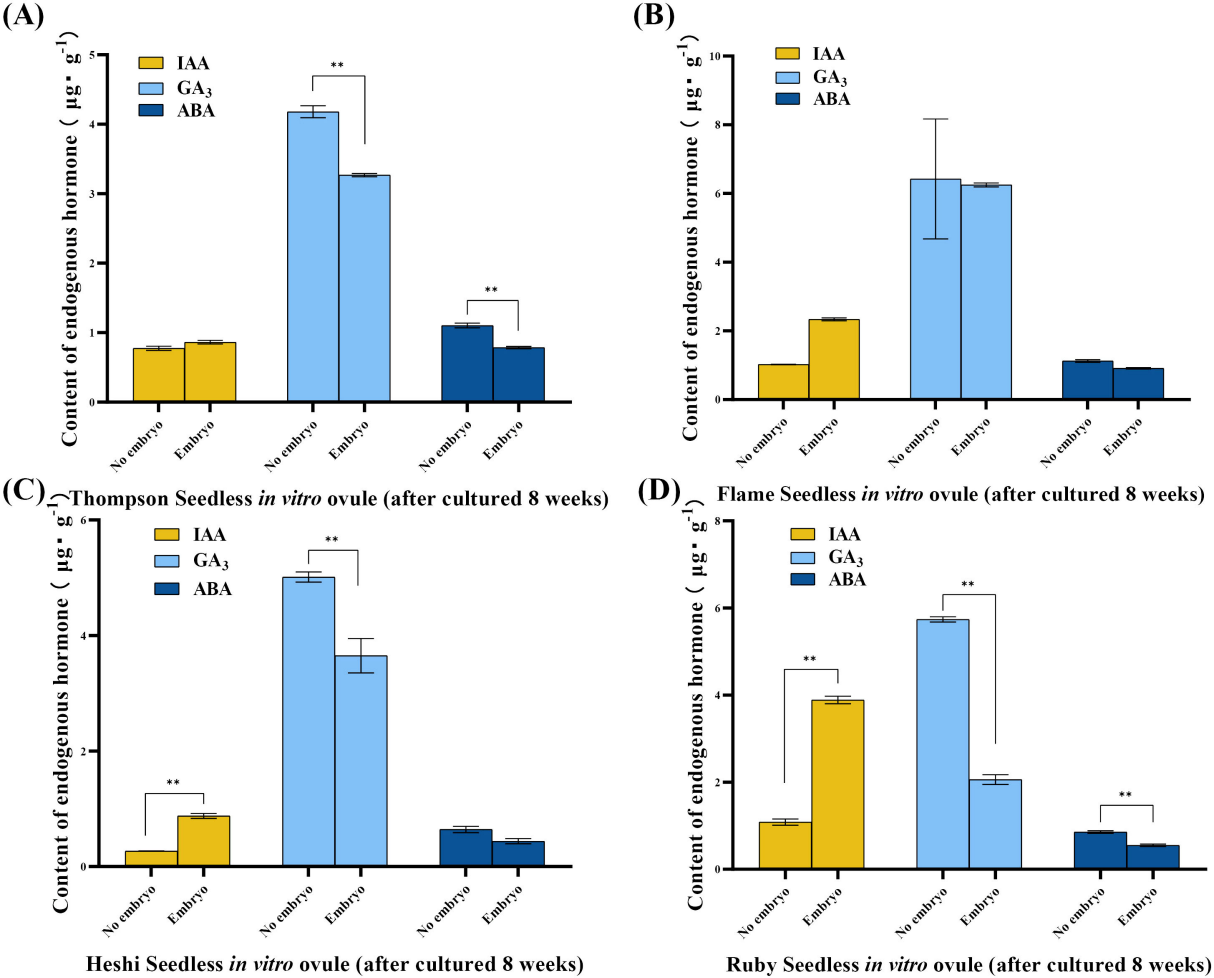
Endogenous hormone levels of different samples of four seedless grape *in vitro* ovules (after 8 weeks of culture). **(A)** “Thompson Seedless” grape *in vitro* ovules. **(B)** “Flame Seedless” grape *in vitro* ovules. **(C)** “Heshi Seedless” grape *in vitro* ovules. **(D)** “Ruby Seedless” grape *in vitro* ovules. The data are presented as mean ± SD, *n* = 3. ^**^ *P* < 0.01 indicates significantly different values.

IAA level: The significance analysis showed that the IAA level was significantly higher in ovules with embryos than in those without embryos. The analysis indicated that the most suitable embryo rescue time for these four seedless grapes was considered. The IAA level was higher in *in vitro* ovules with developing embryos than in those without embryos after 8 weeks of culture under the same *in vitro* conditions. This suggested that the high IAA level promoted the growth and development of embryos in *in vitro* ovules. Conversely, the lower IAA level inhibited the growth and development of the embryo, leading to gradual abortion (**Figure 3**).

GA_3_ level: The statistical analysis suggested that, during the most suitable embryo rescue time, the GA_3_ level was lower in *in vitro* ovules with developing embryos than in those without embryos. This indicated that the high GA_3_ level inhibited the growth and development of embryos *in vitro*, leading to gradual abortion. On the contrary, when the GA_3_ level was low, embryo abortion was reduced (**Figure 3**).

ABA level: The statistical analysis indicated that, during the most suitable embryo rescue time, the ABA level was lower in *in vitro* ovules with developing embryos than in those without embryos. This suggested that the high ABA level might inhibit the growth and development of embryos *in vitro*, leading to gradual abortion. On the contrary, when the ABA level was low, the degree of embryo abortion decreased (**Figure 3**).

### 3.2 Effects of exogenous hormones on ovule development in berries and *in vitro* ovule development in seedless grapes

#### 3.2.1 Effects of spraying exogenous hormones on ovule development in seedless grapes

**Figure 4:**
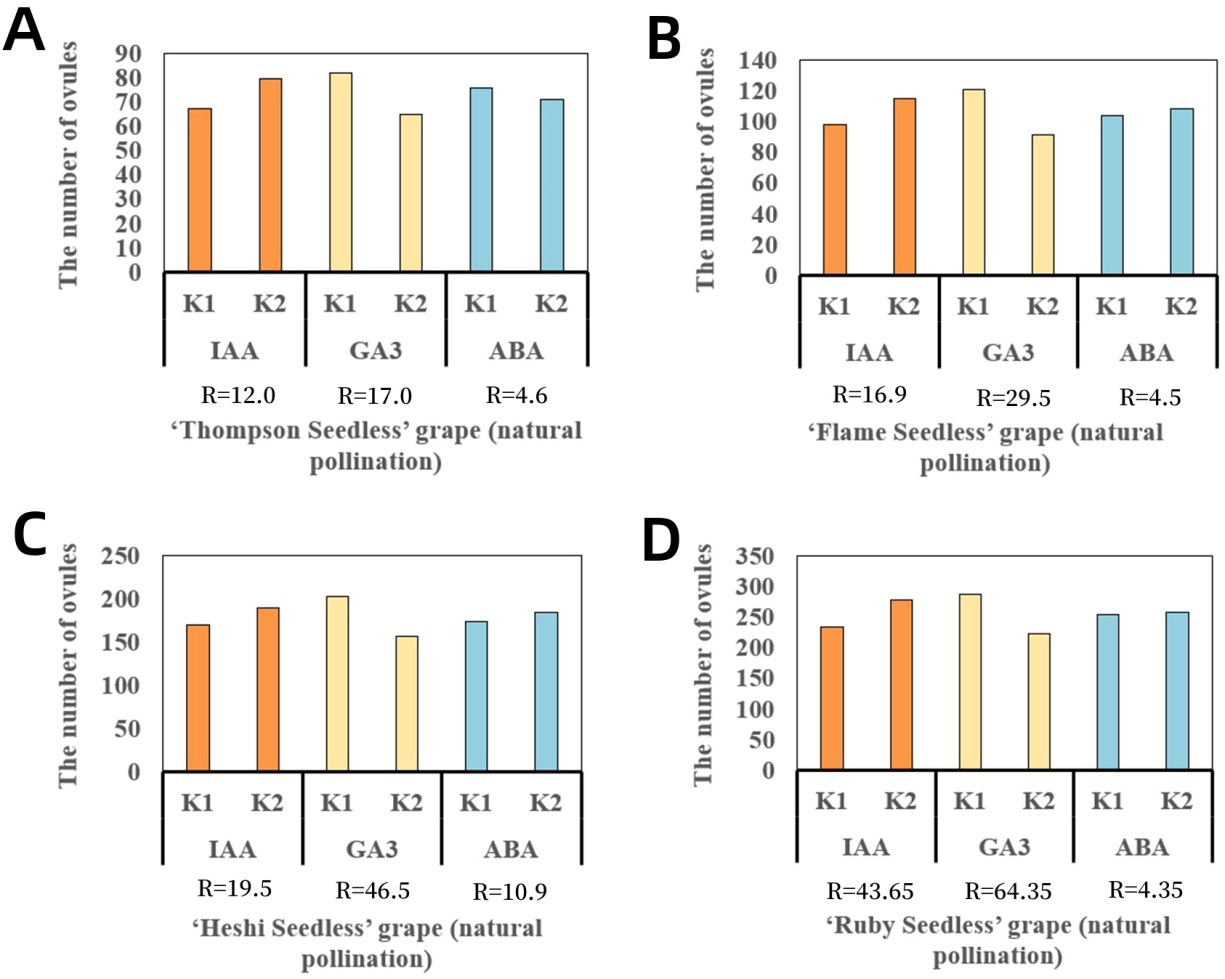
Range analysis of the effect of spraying exogenous hormones on the number of ovules in seedless grapes. **(A)** “Thompson Seedless” grapes. **(B)** “Flame Seedless” grapes. **(C)** “Heshi Seedless” grapes. **(D)** “Ruby Seedless” grapes. K1 represents the average number of ovules of each factor at the first level. K2 represents the average number of ovules of each factor at the second level. R stands for range (between K1 and K2).

When sprayed with different ratios of exogenous hormones, the two levels of IAA in four cultivars of seedless grape berries were 67.5 and 79.5 (“Thompson Seedless”), 97.8 and 114.7 (“Flame Seedless”), 169.5 and 189.0 (“Heshi Seedless”), and 234.0 and 277.7 (“Ruby Seedless”). It was observed that the total number of ovules in each cultivar showed an increasing trend with the increase in the levels of the IAA factor. The two levels of GA_3_ in four cultivars of seedless grape berries were 82.0 and 65.0 (“Thompson Seedless”), 121.0 and 91.5 (“Flame Seedless”), 202.5 and 156.0 (“Heshi Seedless”), and 288.0 and 223.7 (“Ruby Seedless”). The results indicated that the total number of ovules increased with the decrease in the level of the GA_3_ factor. When different ratios of exogenous hormones were used for spraying, the two levels of ABA in the four cultivars of seedless grape berries were 75.8 and 71.2 (“Thompson Seedless”), 104.0 and 108.5 (“Flame Seedless”), 173.8 and 184.7 (“Heshi Seedless”), and 253.7 and 258 (“Ruby Seedless”). It was observed that the total number of ovules increased with the increase in the ABA level. Therefore, based on orthogonal experimental analysis, it was concluded that among the three selected factors, the primary and secondary correlations affecting the total number of ovules in the four seedless grape cultivars were GA_3_, IAA, and ABA. This indicated that spraying GA_3_ significantly impacted the development of ovules in seedless grapes, followed by IAA and ABA (**Table 3**, **Figure 4**).

**Table 3:** Effects of exogenous hormones on ovule development in seedless grapes.

Cultivars	Treatment^a^	Number of bunches	Number of berries	Number of ovules
“Thompson Seedless” (natural pollination)	EH1	5	900	78.3
	EH2	5	900	56.7
	EH3	5	900	85.7
	EH4	5	900	73.3
“Flame Seedless” (natural pollination)	EH1	5	900	110.3
	EH2	5	900	85.3
	EH3	5	900	131.7
	EH4	5	900	97.7
“Heshi Seedless” (natural pollination)	EH1	5	900	187.3
	EH2	5	900	151.7
	EH3	5	900	217.7
	EH4	5	900	160.3
“Ruby Seedless” (natural pollination)	EH1	5	900	264.0
	EH2	5	900	204.0
	EH3	5	900	312.0
	EH4	5	900	243.3

a Treatments of different concentrations of exogenous hormones on seedless grapes were divided into four groups: EH1, EH2, EH3, and EH4 (endogenous hormone abbreviated as EH).

Selection of suitable combinations of exogenous hormones: Upon examining the mean levels of each factor, it was observed that the second level of the IAA factor, the first level of the GA_3_ factor, and the second level of the ABA factor were all the highest. Therefore, the appropriate combination of exogenous hormone level was determined to be IAA2GA_3_1ABA2, indicating that the suitable combination of exogenous hormone ratio was 30 mg·L^–1^ IAA + 30 mg·L^–1^ ABA (**Figure 4**).

#### 3.2.2 Effects of exogenous hormones on the ovule development of seedless grapes cultured *in vitro*

**Figure 5:**
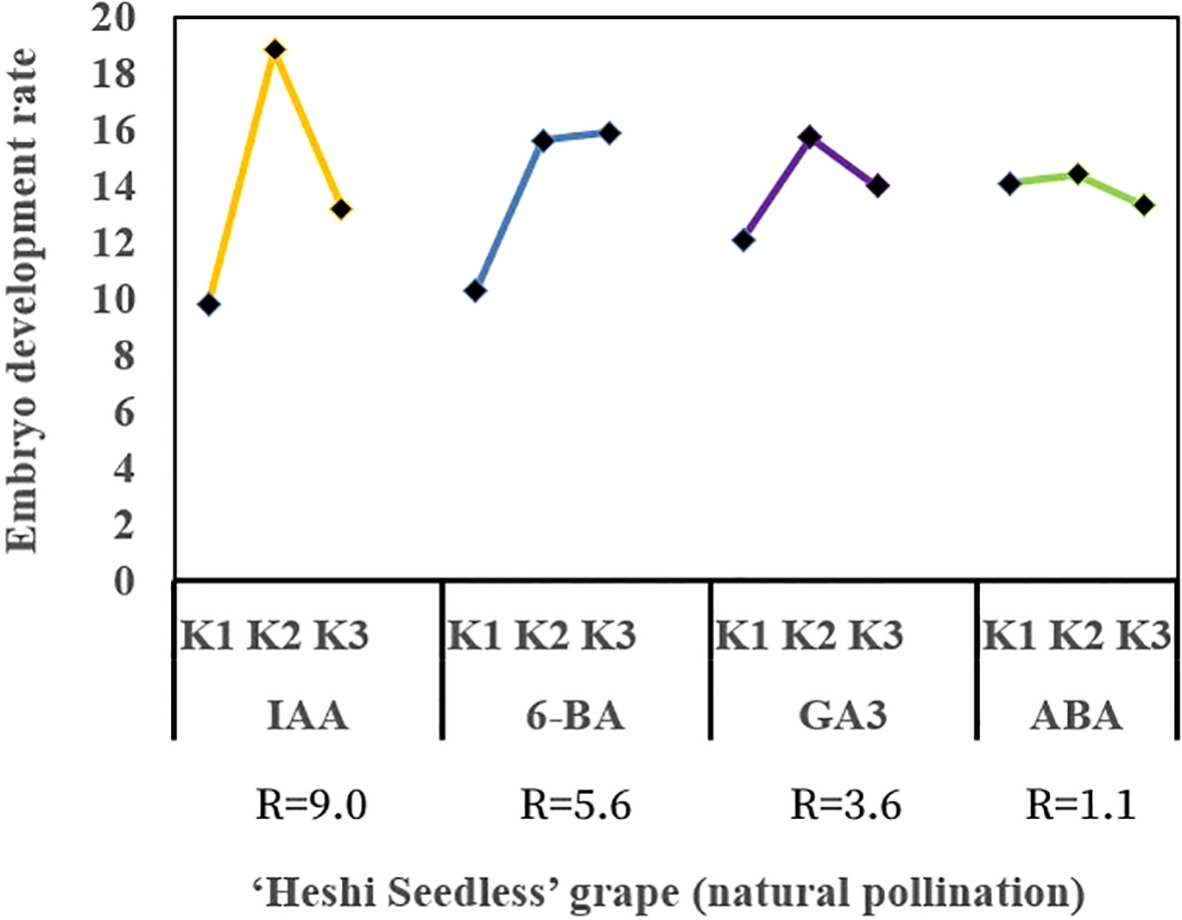
Effects of exogenous hormones on the ovule development in seedless grapes *in vitro*. K1, K2, and K3 represent the average development rate of an embryo of each factor at the first, second, and third levels, respectively. R stands for range (between K1, K2, and K3).

When the ovules of “Heshi Seedless” grapes were cultured *in vitro* with different exogenous hormones, the three levels of the IAA factor were 9.8, 18.8, and 13.2. The results indicated that the embryo development rate of seedless grapes initially increased and then decreased with the increase in the IAA factor. Similarly, for the three levels of the 6-BA factor (10.3, 15.6, and 15.9), an increasing trend in the embryo development rate of seedless grapes was observed with the increase in the level of the 6-BA factor. Regarding the three levels of the GA_3_ factor (12.1, 15.7, and 14.0), an initial increase followed by a decrease in the development rate of seedless grape embryos was noted with an increase in the level of the GA_3_ factor. Likewise, the three levels of ABA factors (14.1, 14.4, and 13.3) showed an initial increase and then a decrease in the development rate of seedless grape embryos with an increase in the ABA factors. Therefore, the orthogonal analysis revealed that IAA, 6-BA, GA_3_, and ABA significantly affected ovule development *in vitro* (**Table 4**, **Figure 5**).

**Table 4:** Effects of exogenous hormones on *in vitro* embryo germination and seedling formation in seedless grapes.

Cultivar	Treatment^a^	Number of ovules cultured	Number of embryos developed	Number of embryos developed	Number of embryos germinated	% of embryos germinated	Number of plantlets developed	% of plantlets developed
“Heshi Seedless”	H1	500	22	4.4	9	40.9	2	9.1
	H2	500	60	12.0	31	51.7	9	15.0
	H3	500	65	13.0	35	53.8	10	15.4
	H4	500	73	14.6	37	50.7	13	17.8
	H5	500	112	22.4	71	63.4	32	28.6
	H6	500	97	19.4	59	60.8	26	26.8
	H7	500	59	11.8	34	57.6	12	20.3
	H8	500	62	12.4	36	58.1	13	21.0
	H9	500	77	15.4	44	57.1	19	24.7

a Treatments of different concentrations of exogenous hormones on the in vitro culture of seedless grapes were divided into nine groups: H1, H2, H3, H4, H5, H6, H7, H8, and H9 (hormone abbreviated as H).

Selection of the suitable combination of exogenous hormones: Based on the average levels of all factors, IAA factor 2, 6-BA factor 3, GA_3_ factor 2, and ABA factor 2 exhibited the highest values. Therefore, the appropriate combination of exogenous hormones was determined to be IAA2BA3GA_3_2ABA2. The optimal ratio of exogenous hormones was 1.0 mg·L^–1^ IAA + 2.0 mg·L^–1^ 6-BA + 1.0 mg·L^–1^ GA_3_ + 1.0 mg·L^–1^ ABA (**Figure 5**).

#### 3.2.3 Effects of exogenous hormones on the germination of embryos and the formation of seedlings in seedless grapes cultured *in vivo*

**Figure 6:**
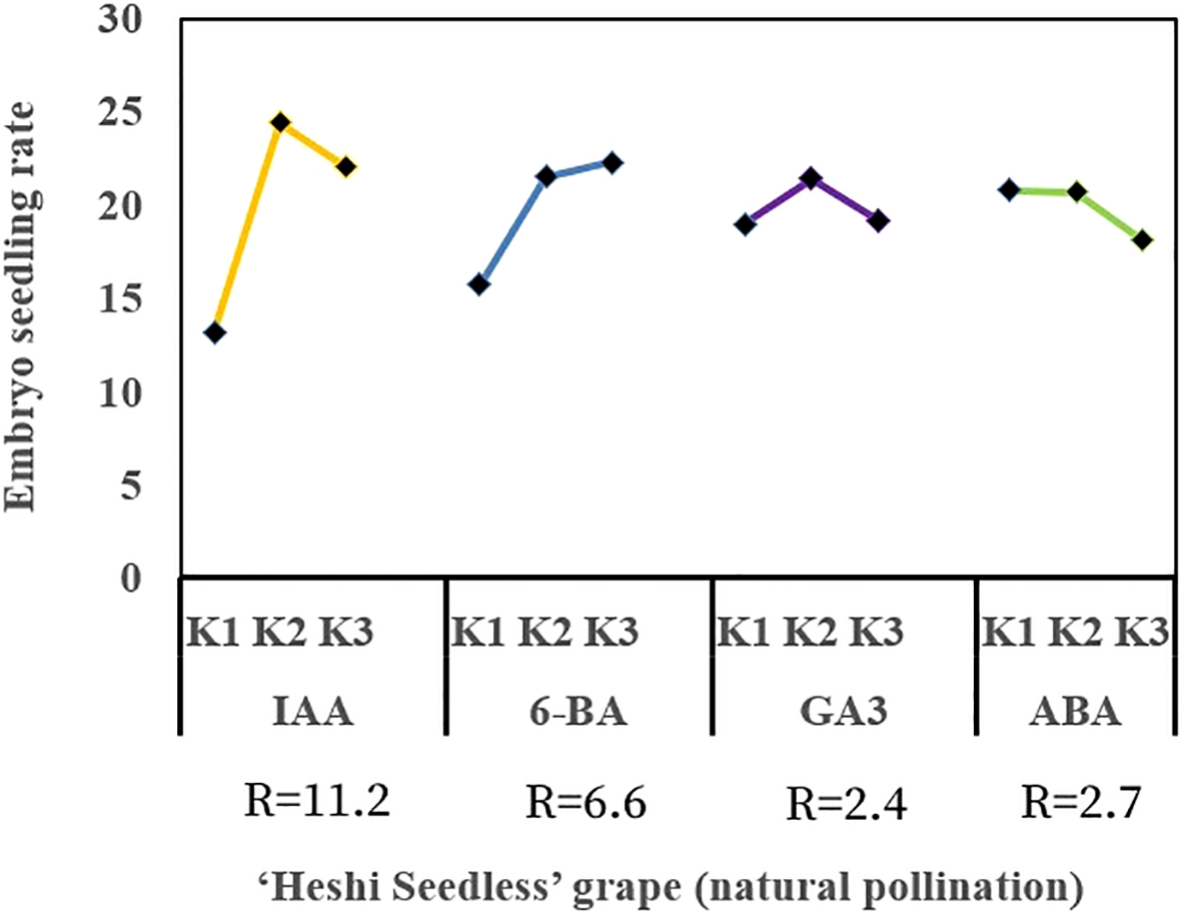
Range analysis of the effect of exogenous hormones on *in vitro* embryo germination and seedling formation in seedless grapes. K1, K2, and K3 represent the average seeding rate of a seedless embryo of each factor at the first, second, and third levels, respectively. R stands for range (between K1, K2, and K3).

When different exogenous hormones were introduced to the embryos of “Heshi Seedless” grapes *in vitro*, the three levels of the IAA factor were 13.2, 24.4, and 22.0, respectively. This indicated that the rate of seedling formation in seedless grape embryos initially increased and then decreased with an increase in the levels of the IAA factor. Similarly, the levels of the 6-BA factor were 15.7, 21.5, and 22.3 indicating an upward trend in the seedling formation rate with increasing levels of the 6-BA factor. In contrast, the three levels of the GA_3_ factor were 19.0, 21.4, and 19.2 indicating that the seedling formation rate initially increased and then decreased with increasing levels of the GA_3_ factor. Finally, the three levels of the ABA factor were 20.8, 20.7, and 18.1 indicating a decrease in the seedling formation rate of seedless grape embryos with increasing levels of the ABA factor. Therefore, based on orthogonal test analysis, the primary and secondary correlations affecting the seedling rate of seedless embryos were identified as IAA, 6-BA, ABA, and GA_3_ (**Table 4**, **Figure 6**).

**Figure 7:**
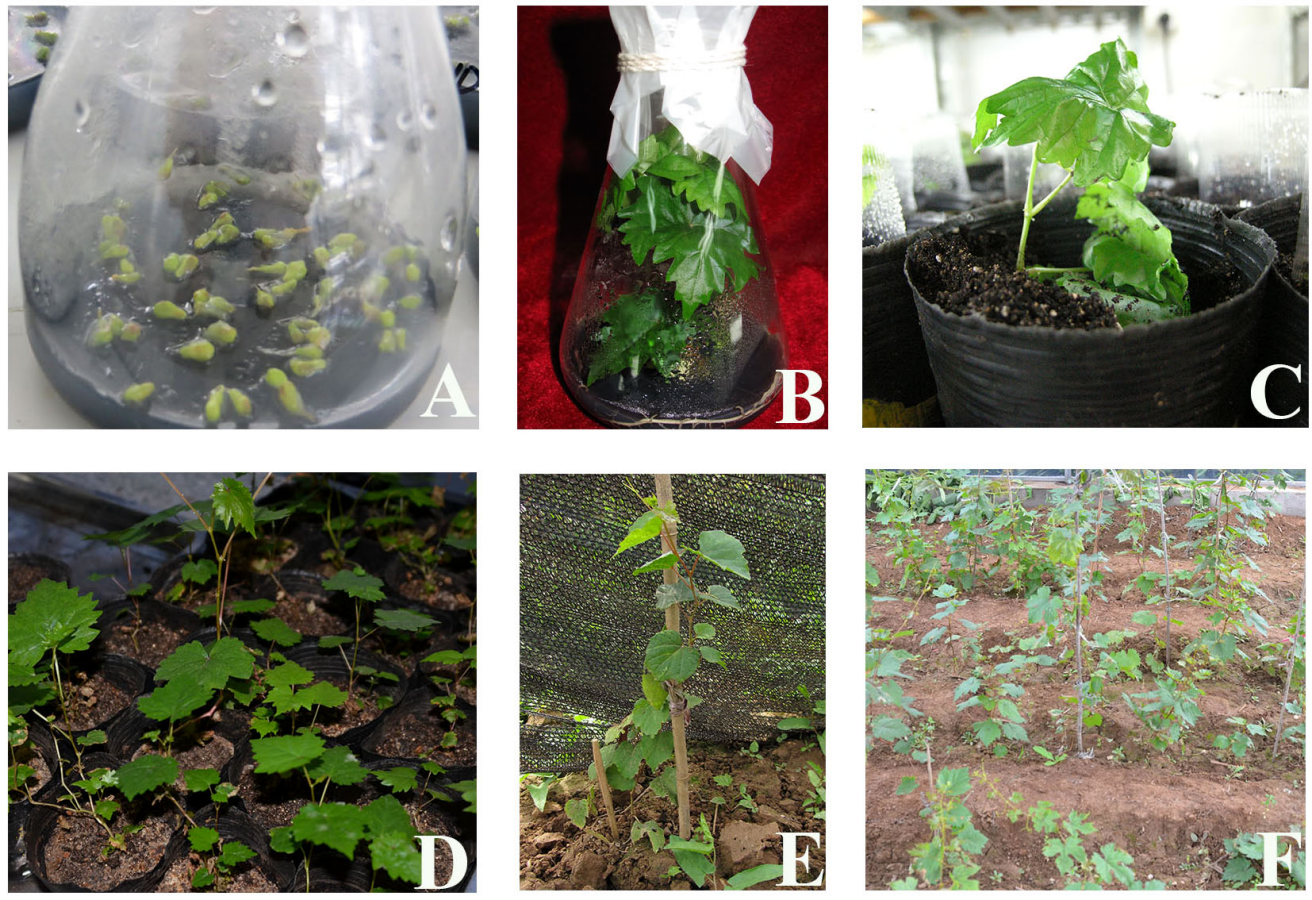
Germination and seedling formation of *in vitro* ovules under the best combination of exogenous hormones. **(A)** *In vitro* culture of ovules. **(B)** Embryo germination into seedlings. **(C)** Seedling training. **(D)** Domestication. **(E, F)** Field transplantation.

Selection of suitable combinations of exogenous hormones: The study found that the second level of the IAA factor, the third level of the 6-BA factor, the second level of the GA_3_ factor, and the first level of the ABA factor exhibited the highest efficacy. Therefore, the appropriate combination of exogenous hormones was determined to be IAA2BA3GA32ABA1, corresponding to 1.0 mg·L^–1^ IAA, 2.0 mg·L^–1^ 6-BA, 1.0 mg·L^–1^ GA_3_, and 0.0 mg·L^-1^ ABA (**Figures 6**, **7**).

## 4 Discussion

### 4.1 Changes in endogenous hormones during the embryonic development of seedless grapes

During the embryonic development of seedless grapes, the changes in endogenous hormone levels are closely associated with the growth and development of ovules and embryos, thus playing a crucial role. Therefore, investigating these hormonal changes during seedless grape embryo development is highly significant for delaying embryo abortion and improving embryo rescue rates.

IAA is a pivotal factor in somatic embryogenesis. It serves as a key factor in inducing somatic embryogenesis (Dudits et al., 1991). Studies have revealed that the emergence of embryonic cells in rice often coincides with elevated endogenous IAA levels. Additionally, the introduction of exogenous IAA or inhibitors preventing IAA efflux during embryonic cell transformation tends to promote the formation of embryonic cells (Chen et al., 1998). This suggests that maintaining or increasing the endogenous IAA level during plant embryonic development is a common requirement for inducing embryogenesis and supporting embryo development. The earliest study on seedless grapes was conducted in 1960 when Nitsh et al. (1960) reported a higher IAA level in the ovary of seedless canker than in the ovary of seeded canker up to 10 days after flowering. However, from 20 days after flowering, the endosperm and embryo of seedless canker degraded and detached, leading to a decrease in the IAA level. Chen et al. (2002) examined the Seedless White, Jufeng, and Muna cultivars, measuring their endogenous hormone levels. They observed significantly higher IAA levels in Seedless White grapes in the early stages of berry development, contributing to fruit setting. During the later stage of berry development, the IAA and GA_3_ levels decreased, whereas the ABA level increased, in Seedless White grapes compared with the other two seedless cultivars. The change in the GA_3_ to ABA ratio plays a key role in berry development. Yan (2006) analyzed the endogenous hormone level in young fruits of Cressen Seedless and Wonderful Seedless grapes, studying their impact on fruit setting in seedless grapes. Pan et al. (2010) assessed IAA, GA_3_, cytokinin, and ABA levels in seedless grape ovules during embryo development and abortion. They found that, in seedless grapes with seed abortion, the ratio of (IAA + GA_3_)/ABA and (ZT + ZRs + KT + iPAs)/ABA was significantly lower in various stages of grape development than in the control group, indicating a close correlation with embryo abortion.

In this experiment, the higher IAA level was observed when the number of ovules in different types of berries was higher. This indicated that the elevated IAA level was conducive to ovule growth and development. Conversely, when ovules were absent, the IAA level was low. This indicated that the reduced IAA level inhibited ovule growth to some extent, resulting in a reduction in the number of ovules until they disappeared. The IAA level was higher in *in vitro* ovules with embryos than in *in vitro* ovules without embryos, suggesting that the high IAA level was conducive to the growth and development of embryonic ovules *in vitro*. Conversely, when the IAA level was low, the growth and development of embryos in this environment were also inhibited to some extent, leading to gradual abortion. It was evident that auxin was one of the key factors in the embryo development and embryo rescue process of seedless grapes. Relevant theoretical research has thus provided a foundation for enhancing seedless grape embryo rescue breeding technology.

Studies on the role of GA_3_ in regulating somatic embryogenesis and development are limited. Xing et al. (1999) investigated the changes in the gibberellin level during the somatic embryogenesis of Crown grass and found that the trend of GA_3_ change was similar to that of the changes in the cytokinin level, suggesting the potential involvement of GA_3_ in regulating cell growth. However, most plants can produce somatic embryos in the medium without exogenous GA_3_, indicating that endogenous GA_3_ may also affect somatic embryogenesis. GA_3_ is commonly used in seedless grape production and cultivation, and also in the seedless treatment technology of grapes. Lu and Lamikanra (1996) suggested that externally applying GA_3_ to treat seedless grapes might result in the production of some seedless fruits, indicating a correlation between GA_3_ and embryonic development.

In this experiment, when the number of ovules in seedless grapefruit was high, the GA_3_ level was low. This indicated that the high GA_3_ level was unfavorable for the growth and development of ovules in berries. Conversely, when no ovule was present, the GA_3_ level was higher. This suggested that the high GA_3_ level in berries inhibited ovule growth to some extent, leading to a reduction in the number of ovules until they disappeared. Moreover, in the case of a developing embryo in the isolated ovule, the GA_3_ level in the ovule was lower than that in the ovule without an embryo. This indicated that the high GA_3_ level in the ovule inhibited the growth and development of embryos, eventually leading to its abortion, which was detrimental to the growth and development of embryos. Conversely, when the GA_3_ level was low, embryo abortion reduced. These findings suggested a correlation between the GA_3_ level and embryo abortion in seedless grapes, providing a theoretical basis for using GA_3_ spray to induce seedlessness in grapes.

ABA plays a crucial role in plant embryogenesis and development. It promotes the initiation of plant embryos. Taber et al. (1998) classified the somatic embryonic development of Sequoia Fortunei into three stages: maintenance, ABA stimulation, and maturation. Han and Li (1993) observed that, during the formation of carrot somatic embryos, the cells that formed embryos had higher ABA levels than those that did not. ABA also promotes the maturation of somatic embryos, inhibits premature germination, and suppresses the formation of abnormal embryos (Cui et al., 2000). However, some studies have shown that ABA can inhibit embryo formation (Walker-Simmons, 1987). The impact of ABA on embryonic development is multifaceted. (1) High concentrations of ABA appear inhibitory, whereas suitable low concentrations promote embryo formation. (2) Different plant species exhibit varying responses to different ABA levels. Wang et al. (1992) analyzed the endogenous ABA level in grapes and seedless grapes, noting that the ABA level in seedless grapes was consistently higher. In addition, the trend of the changes in the endogenous ABA level in these grapes was similar. Kondo and Kawai (1998) observed that the endogenous ABA level was significantly lower in seeded Pione grapes than in seedless Pione grapes. He et al. (2013) found that from 10 to 30 DAS (Changes in fruit volume over time followed a “S” pattern from 10 to 60 days after fruit set), fruits with aborted embryos exhibited lower IAA and GA_3_ levels but higher ABA levels compared with fruits with normal embryos. Additionally, the ratios of ABA/ZT and ABA/(GA_3_ + IAA + ZT) were higher in fruits with aborted embryos. The IAA/GA_3_ ratio was positively correlated with fruit size. These findings suggested that the higher endogenous ABA level during embryo abortion promoted embryo abortion.

In this study, when seedless grapes of different berry types had a high number of ovules, the ABA level was low, indicating that the high ABA level was not conducive to the growth and development of ovules in berries. Conversely, when no ovules were present, the ABA level was higher, suggesting that the high ABA level in berries inhibited ovule growth to some extent, leading to a decrease in the number of ovules until they disappeared. Regarding *in vitro* ovules with or without embryos, when developmental embryos were present, the ABA level was lower in the *in vitro* ovules with embryos than in those without embryos. This indicated that the growth and development of embryos *in vitro* were inhibited in an environment with a higher ABA level, resulting in gradual abortion, which was unfavorable for embryo development. Conversely, the lower ABA level reduced the degree of embryo abortion. These findings suggested a close correlation between these three endogenous hormones and seedless grape ovule development. The decrease in the IAA level and the increase in GA_3_ and ABA levels might contribute to a reduction in the number of ovules until they disappeared.

### 4.2 Effects of exogenous hormones on embryo rescue and seedling formation in seedless grapes

During the development of seedless grapes, it is crucial to investigate whether the application of exogenous hormones impacts endosperm development, alleviates embryo abortion, and increases embryo rescue efficiency. Tang et al. (2009a) observed a positive effect of the exogenous hormone Cycocel (CCC) on ovule development. Some studies indicated that an excess of exogenous hormone ABA affected ovule growth during fruit development (Chen and Lu, 2000; Finkelstein et al., 1985). In addition, studies found that the proportion and balance of plant hormones during exogenous hormone application were crucial for preventing embryo abortion during fruit development compared with individual hormones (Li et al., 2020b). This study explored the balance between multiple exogenous hormones using an orthogonal experimental design. The study revealed that the optimal combination of exogenous hormones for seedless grape ovule development was 30 mg·L^–1^ IAA + 30 mg·L^–1^ ABA. These findings demonstrated that exogenous hormones could promote the development of inner ovules in the fruits and *in vitro* ovules of seedless grapes, mitigate embryo abortion, and increase the embryo rescue rate.

The effects of adding exogenous hormones to the culture medium vary during embryo rescue in seedless grapes. Some argue that seedlings can develop without adding exogenous hormones. Raghavan (1966) suggested that immature embryos reaching a certain stage could be autotrophic and synthesize hormones independently, obviating the need for exogenous hormones. Although some errors were observed in the cytokinin level in seedless grapes and *in vitro* ovules in the early stage of this study, no further analysis was conducted. However, the data showed that the addition of 6-BA during the embryo rescue of seedless grapes promoted *in vitro* ovule development and embryo germination (Bordelon and Moore, 1994; Agüero et al., 2000; Valerii et al., 2002, 2005). Therefore, in the embryo rescue seedling pre-experiment, 6-BA was added and shown to impact embryo germination. In this study, four exogenous hormones were selected for addition to the culture medium to examine their effects on *in vitro* embryo development and embryo germination into seedlings during embryo rescue in seedless grapes. The results revealed that the optimal combination of exogenous hormones for *in vitro* embryo development was 1.0 mg·L^–1^ IAA + 2.0 mg·L^–1^ 6-BA + 1.0 mg·L^–1^ GA_3_ + 1.0 mg·L^–1^ ABA. Similarly, the optimal combination for *in vitro* embryo germination into seedlings was 1.0 mg·L^–1^ IAA + 2.0 mg·L^–1^ 6-BA + 1.0 mg·L^–1^ GA_3_ + 0.0 mg·L^–1^ ABA. Spiegel-Roy et al. (1985) investigated the effects of exogenous hormones on the rescue of embryos in seedless grapes and found that a combination of 10^–5^μM and 10^–5^μM was optimal for ovule germination and seedling development. Similarly, Tian et al. (2008) reported that adding GA_3_ and IAA significantly increased the embryo rescue rate of Emerald Seedless × Beichun. These findings were consistent with the results of Spiegel-Roy et al. (1985) and Tian et al. (2008), indicating that adding specific exogenous hormones was beneficial for the development of *in vitro* ovules and embryo germination into seedlings.

In the future, we will continue to conduct systematic research and analysis of the changes in the endogenous cytokinin level in seedless grapes and *in vitro* ovules, as well as the impact of exogenous cytokinin on embryo rescue. Exploring and improving the equilibrium among various exogenous hormone levels, refining the composition of the embryo rescue culture medium, and improving the embryo rescue rate in seedless grapes are crucial. This study aimed to provide suitable embryo rescue breeding technologies and novel germplasm for seedless grape cultivars.

## 5 Conclusion

This study elucidated the pivotal role of hormonal changes during fruit development and *in vitro* ovule development in seedless grapes, influencing the progression and termination of immature embryos. The four endogenous hormones were closely linked to embryo rescue in seedless grapes. A decrease in the IAA level and an increase in GA_3_ and ABA levels led to a decrease in the number of ovules, potentially culminating in their disappearance. Proper supplementation of 6-BA proved beneficial for embryo germination. Different exogenous hormones exerted varying effects on embryo rescue. An optimal combination significantly enhanced ovule growth and development, yielding higher rates of embryo development and seedling formation. This study enriched our understanding of the interaction between hormones and embryonic development, providing a theoretical basis for improving embryo rescue rates and exploring strategies to bolster the efficiency of embryo rescue in seedless grapes. Furthermore, this study offered hybrid germplasm resources for the breeding of new seedless grape cultivars, facilitating future cultivar improvement and optimization. The intricate correlation between polyamines and hormones in embryo abortion warrants further investigation to achieve deeper insights into the seedless grape embryo rescue mechanism and promote embryo rescue breeding in seedless grapes.

## Data Availability

The original contributions presented in the study are included in the article/supplementary material. Further inquiries can be directed to the corresponding author.
